# Impact of immune checkpoint inhibition on ovarian reserve

**DOI:** 10.1093/oncolo/oyag048

**Published:** 2026-02-28

**Authors:** Elizabeth I Buchbinder, Zihe Song, Justine V Cohen, Sandra J Lee, Kimberly K Smith, Michael Manos, Ahmad Tarhini, Michael Dougan

**Affiliations:** Department of Medical Oncology, Dana-Farber Cancer Institute, Boston, MA 02215, USA; Division of Biostatistics, Department of Data Science, Dana-Farber Cancer Institute, Boston, MA 02215, USA; Department of Medical Oncology, Dana-Farber Cancer Institute, Boston, MA 02215, USA; Division of Biostatistics, Department of Data Science, Dana-Farber Cancer Institute, Boston, MA 02215, USA; Department of Obstetrics/Gynecology, Brigham and Women’s Hospital, Boston, MA 02215, USA; Department of Medical Oncology, Dana-Farber Cancer Institute, Boston, MA 02215, USA; Department of Medical Oncology, Moffitt Cancer Center, Tampa, FL 33612, USA; Department of Gastroenterology, Massachusetts General Hospital, Boston, MA 02114, USA

**Keywords:** immune checkpoint inhibition, ovarian reserve, melanoma

## Abstract

Immune checkpoint inhibition (ICI) has become a mainstay of therapy for various types of malignancy and is being used earlier in the course of therapy. In addition, there are many young women diagnosed with cancers for whom ICI is the standard of care. However, the impact of ICI on fertility is unknown and hard to assess. To evaluate this, we performed an analysis of hormones associated with ovarian reserve in young women with melanoma treated with ipilimumab. Our study showed a reduction in anti-Mullerian hormone, a marker of ovarian reserve, in patients with melanoma treated with either ICI or targeted therapy. These results support the need for further work in this area to better understand the impact these therapies have on fertility.

Immunotherapy with immune checkpoint inhibition (ICI) is being widely adopted in the treatment of patients with a wide range of malignancies. Despite the first approval of these agents for metastatic melanoma more than a decade ago, and considering approximately 20% of people with melanoma treated with ICI (including ipilimumab, nivolumab, and pembrolizumab) are women of childbearing age, data on how these therapies influence fertility (specifically ovarian reserve) or the likelihood of responding to fertility treatments after therapy are largely lacking.^1^^,^^2^

To investigate ovarian reserve, we obtained pre- and post-treatment serum samples to be analyzed for luteinizing hormone (LH), follicle-stimulating hormone (FSH), estradiol, anti-Mullerian hormone (AMH), and prolactin. The patients were women without pre-existing ovarian or other reproductive pathology aged 20-35 who underwent melanoma treatment with 3 mg/kg ipilimumab (57%) or 10 mg/kg ipilimumab (43%) in the adjuvant setting on trial ECOG-ACRIN E1609. We also obtained serum from two timepoints in 8 patients diagnosed with melanoma who received targeted therapy with dabrafenib and trametinib, as a control group.

Anti-Mullerian hormone is a biomarker of ovarian reserve and a predictor of response to fertility treatments, including in vitro fertilization. In people without ovarian or other reproductive pathology under the age of 35, AMH should be detectable and remain relatively stable over time. Above the age of 35 AMH decline accelerates as the number of eggs decreases more rapidly due to normal ovarian aging. In a study of 366 health care workers aged 21-41 years, the decline in AMH over time was minimal (average of 5.6% of one year).^3^ Based upon this we selected women under the age of 35 for this analysis.

Samples were available from 28 female patients with a median age of 28 (min 20, max 35). The median time between pre- and post-treatment samples was 8.0 months (min 0.7, max 16.4). Fifty-three percent of patients had stage IIIB melanoma, 43% stage IIIC and one patient had M1a disease. Four of the 29 patients experienced treatment-related adrenal insufficiency, one patient experienced hyperthyroidism, one patient experienced hypothyroidism, and three patients experienced other endocrine disorders. At the time of data cutoff 16 patients remain alive, 7 died, 1 refused follow-up, and 4 were officially lost to follow-up.

The median AMH was 4.24 ng/mL in the pre-treatment group and 3.51 in the post-treatment group (Figure 1). Estradiol and LH decreased as well (*P* < .001, *P* = .016, *P* = .012, respectively); levels of FSH and prolactin were similar before and after the treatment. For the control group there were adequate samples available for 8 patients with a median age of 28 years old. The level of AMH dropped significantly (*P* = .046); however, there were no significant changes between levels of Estradiol, FSH, LH, and Prolactin before and after treatment (*P* = .89, .56, .99, .88).

**Figure 1:**
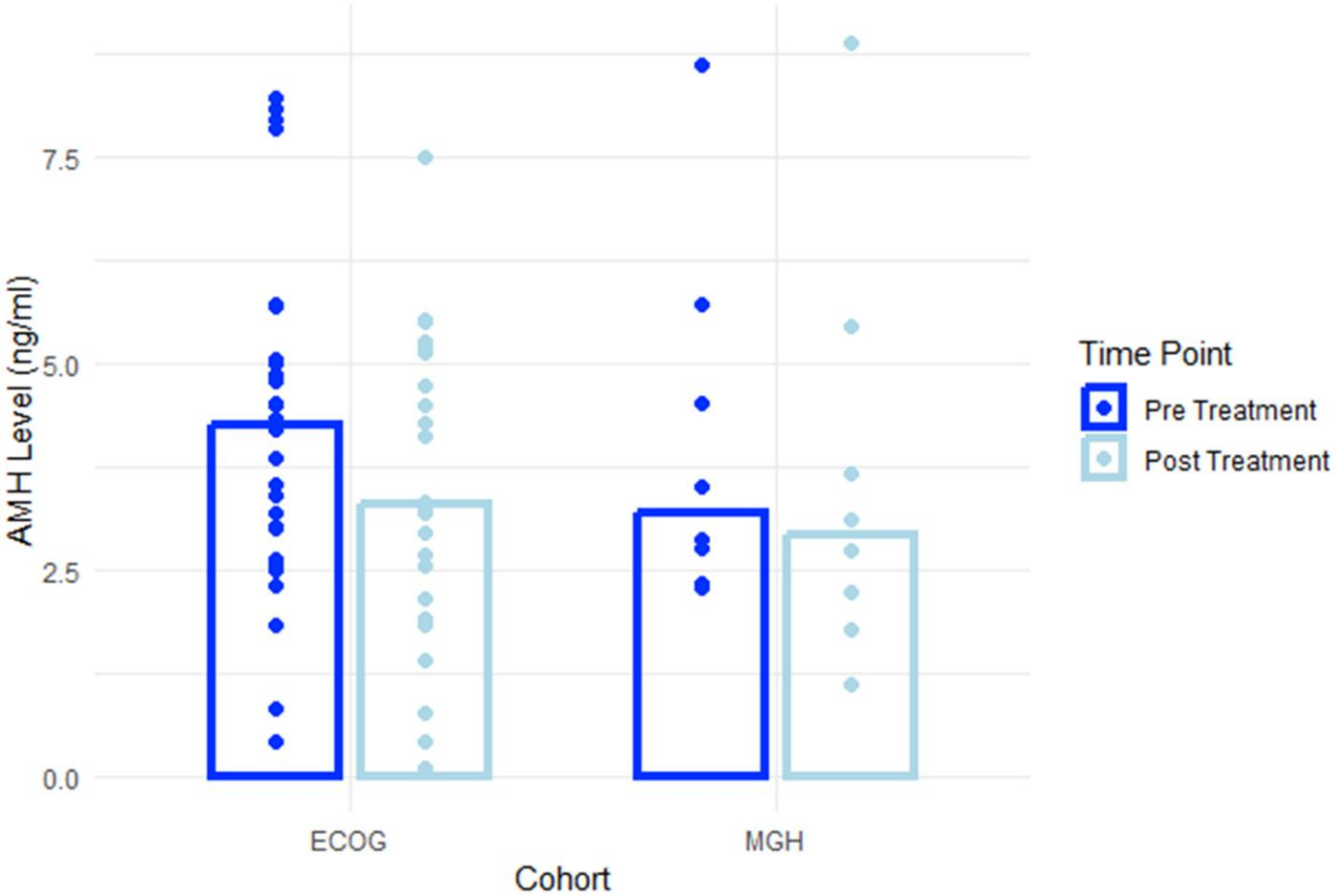
Change of level of AMH before and after treatment (*P *< .001) in patients treated with Ipilimumab (ECOG-ACRIN) and targeted therapy (MGH).

In this study of young women treated with ipilimumab, there was a substantial decrease in AMH after ICI therapy, suggesting that ovarian reserve and possibly female fertility may be impacted by ICI. However, there were similar findings in a small control cohort of melanoma patients treated with targeted therapy. This suggests that the reductions observed might be related to melanoma diagnosis or both immunotherapy and targeted therapy. While ipilimumab alone is rarely used and these findings are not generalizable to all ICI, the similar pattern of toxicities across different ICI still makes this initial study valuable. These findings underscore the importance of further research, in particular in patients treated with PD-1 inhibition, to assess the effects of cancer immunotherapy on fertility more directly, including assessments of the effects of PD-1 inhibition and combination immunotherapy. In addition the impact of cancer immunotherapy-induced endocrine toxicities needs to be explored further.

## Data Availability

Data are available on reasonable request. All data relevant to the study are included in the article or uploaded as online supplemental information. The data generated and/or analyzed during this study are available from the corresponding author on reasonable request.
